# Supplementary material for: Metabolic Innovations Underpinning the Origin and Diversification of the Diatom Chloroplast
Source: Biomolecules. 2019 Jul 30;9(8):322. doi: 10.3390/biom9080322 (PMC6723447; doi:10.3390/biom9080322)
Supplement: Supplementary file 1 [file biomolecules-09-00322-s001.zip › supporting material/Diatom fig. S4.pdf]

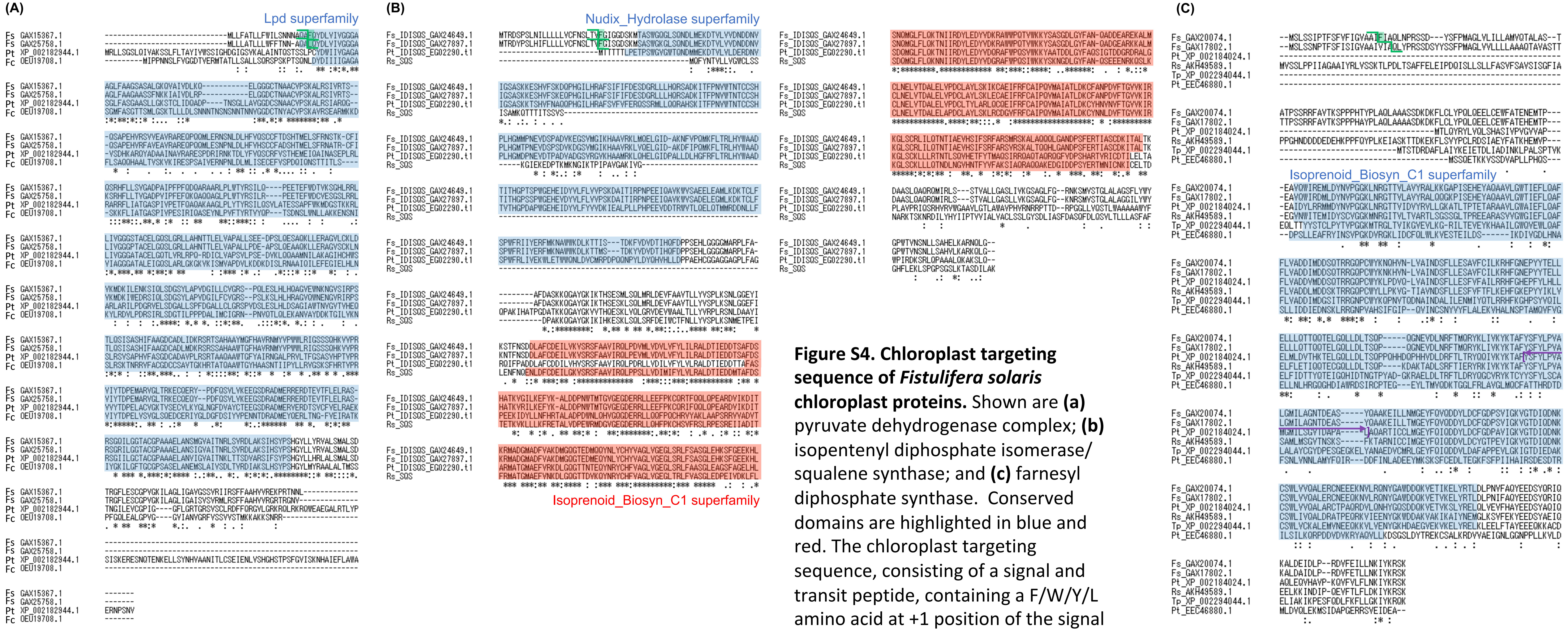

Figure S4. Chloroplast targeting sequence of *Fistulifera solaris* chloroplast proteins. Shown are **(a)** pyruvate dehydrogenase complex; **(b)** isopentenyl diphosphate isomerase/ squalene synthase; and **(c)** farnesyl diphosphate synthase. Conserved domains are highlighted in blue and red. The chloroplast targeting sequence, consisting of a signal and transit peptide, containing a F/W/Y/L amino acid at +1 position of the signal peptide [38], are highlighted in green. Predicted transmembrane regions are shown with violet arrows.
