# Supplementary material for: Metabolic Innovations Underpinning the Origin and Diversification of the Diatom Chloroplast
Source: Biomolecules. 2019 Jul 30;9(8):322. doi: 10.3390/biom9080322 (PMC6723447; doi:10.3390/biom9080322)
Supplement: Supplementary file 1 [file biomolecules-09-00322-s001.zip › supporting material/Diatom fig. S3.pdf]

Fig. S3

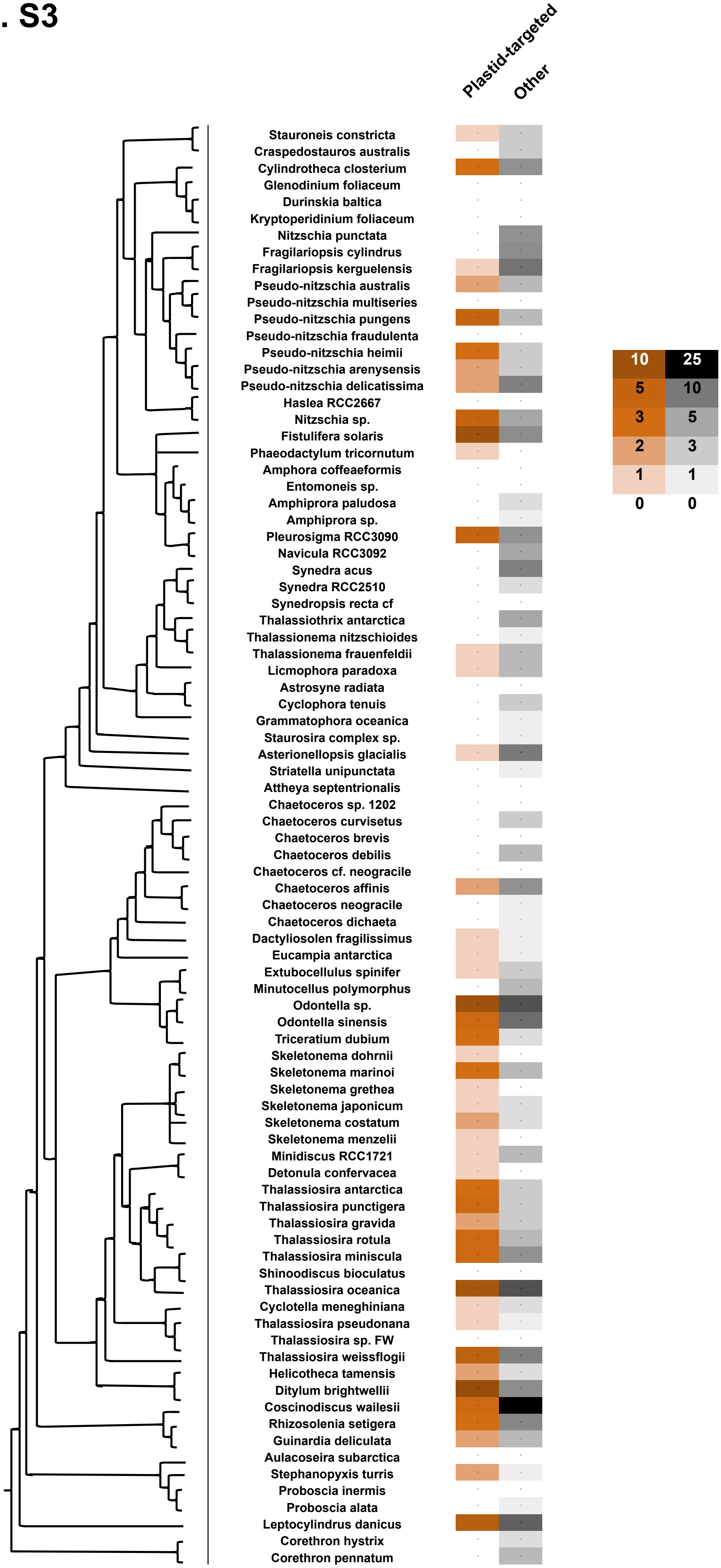

Figure S3. Evolutionary distributions of diatom iota-carbonic anhydrases. This figure shows the number of evolutionarily non-redundant iota-carbonic anhydrases, with inferred chloroplast targeting sequences or otherwise, identified by reciprocal BLAST search of the characterised *T. pseudonana* protein (Thaps_264181), using similar methodology to Fig. S3. Cells are shaded according to the number of copies of proteins identified.
