# Supplementary material for: Metabolic Innovations Underpinning the Origin and Diversification of the Diatom Chloroplast
Source: Biomolecules. 2019 Jul 30;9(8):322. doi: 10.3390/biom9080322 (PMC6723447; doi:10.3390/biom9080322)
Supplement: Supplementary file 1 [file biomolecules-09-00322-s001.zip › supporting material/Diatom fig. S2.pdf]

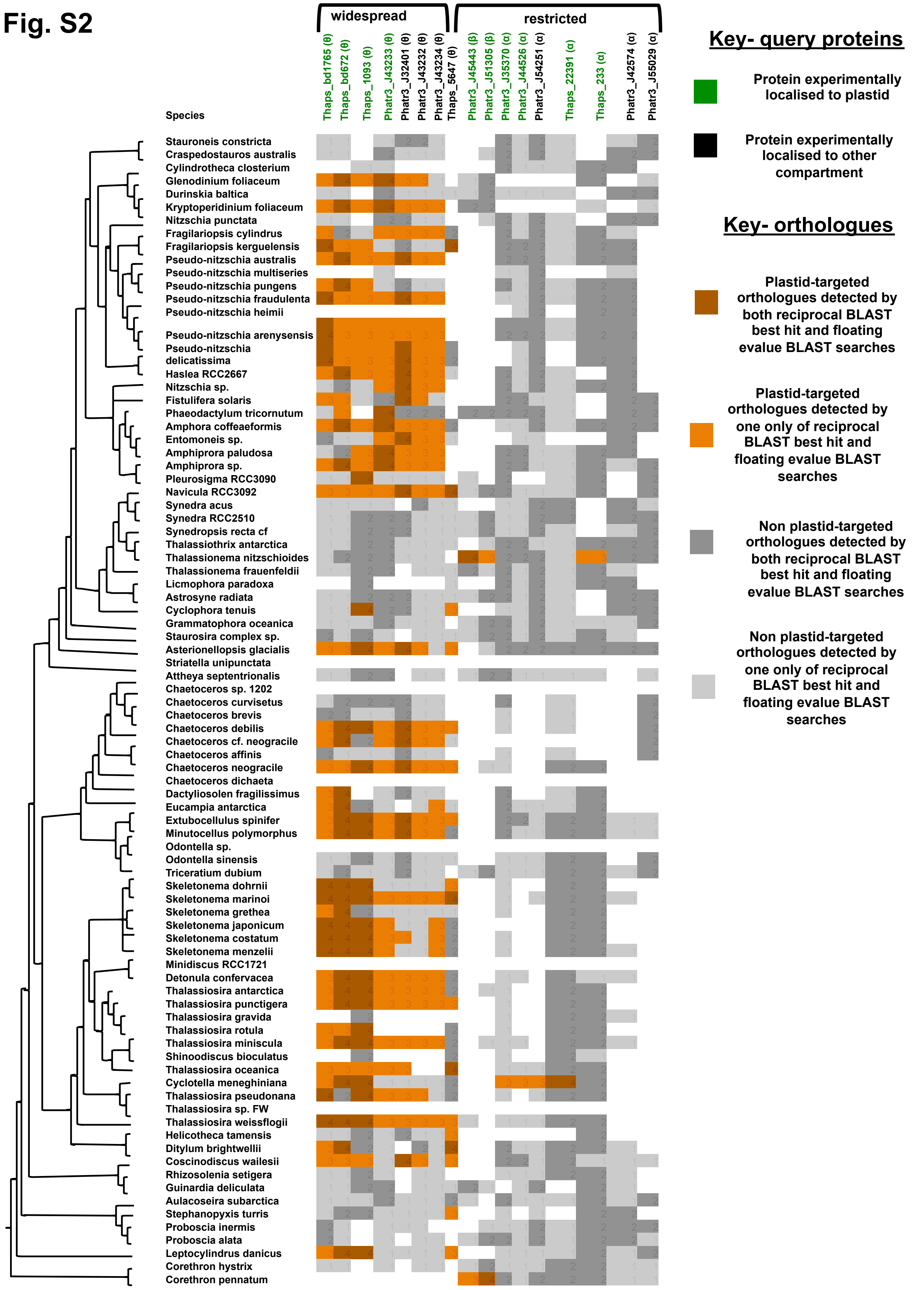

Figure S2. Evolutionary distribution and localisation of diatom carbonic anhydrases. This figure shows the evolutionary distribution of orthologues of experimentally localised carbonic anhydrases from the model diatoms *P. tricornutum* and *T. pseudonana* [41, 72, 98], as inferred by reciprocal BLAST best hit and floating threshold evalue BLAST searches. Query proteins are labelled per structural type of carbonic anhydrase (alpha, beta, or theta); and their experimental localisation (chloroplast, or other compartments e.g., cytoplasm, endomembrane system, and mitochondria). Orthologoues are shaded according to their inferred localisation, based on *in silico* prediction, and whether they are detected by both or only one of the two BLAST strategies employed. Diatom species are ordered following previously published phylogenies [26]. Further details on the carbonic anydrases identified are provided in Table S2.
