# Supplementary material for: Metabolic Innovations Underpinning the Origin and Diversification of the Diatom Chloroplast
Source: Biomolecules. 2019 Jul 30;9(8):322. doi: 10.3390/biom9080322 (PMC6723447; doi:10.3390/biom9080322)
Supplement: Supplementary file 1 [file biomolecules-09-00322-s001.zip › supporting material/Diatom fig. S1.pdf]

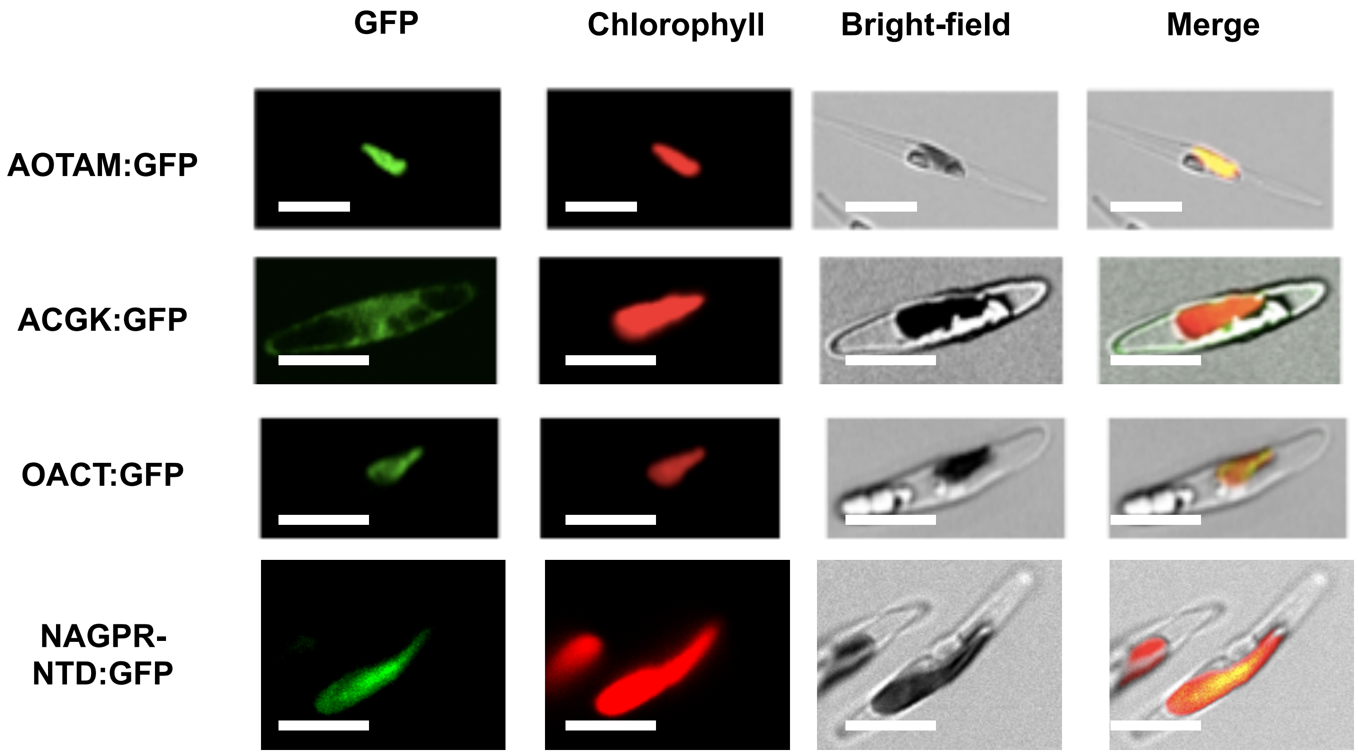

Figure S1. Experimental localisations of diatom ornithine cycle enzymes. This figure shows *P. tricornutum* cell lines expressing C-terminal GFP fusions of enzymes implicated in the chloroplast ornithine cycle : AOTAM (acetylornithine transaminase) ; ACGK (acetylglutamate kinase) ; OACT (ornithine acetyltransferase) ; and NAGPR (N-acetyl-gamma-phosphate reductase), using methodology as per [21]. Constructs labelled « NTD » were made with the N-terminal targeting sequence and first 15 aa of the conserved domain of the protein only; all other constructs were made with full-length protein constructs. Scale bars are to 10 µm.
